# Wave induced coastal flooding along the southwest coast of India during tropical cyclone Tauktae

**DOI:** 10.1038/s41598-022-24557-z

**Published:** 2022-11-19

**Authors:** Ratheesh Ramakrishnan, P. G. Remya, Anup Mandal, Prakash Mohanty, Prince Arayakandy, R. S. Mahendra, T. M. Balakrishnan Nair

**Affiliations:** 1 Space Applications Centre (SAC), ISRO, Ahmedabad, India; 2 Indian National Centre for Ocean Information Services (INCOIS), Ministry of Earth Sciences, Govt. of India, Hyderabad, India; 3 Fisheries Engineering, Kerala University of Fisheries and Ocean Studies (KUFOS), Kochi, Kerala India

**Keywords:** Ocean sciences, Physical oceanography

## Abstract

The coastal flood during the tropical cyclone Tauktae, 2021, at Chellanam coast, Kerala, India, has invited wide attention as the wave overtopping severely affected coastal properties and livelihood. We used a combination of WAVEWATCHIII and XBeach to study the coastal inundation during high waves. The effect of low-frequency waves and the rise in the coastal water level due to wave setup caused the inundation at Chellanam, even during low tide with negligible surge height. Wave setup raised the water level at the coast with steep slopes to more than 0.6 m and peaked during low tide, facilitating wave breaking at the nearshore region. The coastal regions adjacent to these steep slopes were subjected to severe inundation. The combined effect of long and short waves over wave setup formed extreme wave runups that flooded inland areas. At gently sloping beaches, the longwave component dominated and overtopped the seawalls and damaged households along the shoreline. The study emphasizes the importance of longwave and wave setup and its interaction with nearshore bathymetry during the high wave. The present study shall lead to the development of a coastal inundation prediction system for the low-lying hot spots using the combination of WAVEWATCHIII and XBeach models.

## Introduction

Climate change imposes diverse adverse impacts on coastal areas worldwide. Presently, intense cyclones, sea-level rise, storm surges, and extreme waves in the changing climate are the leading causes of coastal vulnerability problems in most coastal regions across the globe^1^. The unprecedented urbanization rate in coastal areas, especially in developing countries, makes coastal vulnerability a serious concern^2,3^. India has a vast coastline covering nine states, and most of these coastal states are densely populated^4^. One of the severe threats to these coastal areas is the intense tropical cyclones and associated coastal flooding and damage^4^.

The Indian Ocean is one of the world’s six cyclone-prone areas^5^. The occurrence of an average of 5–6 intense cyclones per year is expected in the North Indian Ocean (NIO). In the NIO region, cyclone occurrence has been high in the Bay of Bengal (BoB) compared to the Arabian Sea (AS), with an occurrence ratio of 4:1 until the recent past^6^. Recently this ratio has changed mainly because of the rapid warming of the AS, which supports cyclone formation, another visible impact of climate change. The AS started witnessing more intense tropical cyclones (a 150% increase during the last two decades), making India’s west coast vulnerable to cyclones imposing threats like storm surges and high waves^7^. Until recently, the west coast was least prepared for severe cyclones. From Very Severe Cyclone Storm (VSCS) Okhi onwards, the coast experienced the worst damage along the western coastal regions. This was not different in the case of VSCS Tauktae (hereafter referred to as TC Tauktae) in May 2021. The cyclone caused severe damage to many coastal regions as the cyclone traversed parallel to the west coast. High waves were lashing on the coastal areas, which posed a severe threat to the life and property of the coastal population along the west coast until it made landfall in Gujarat on May 17, 2021. In both cases, one of the most affected states was Kerala.

TC Tauktae caused widespread damage in Kerala, especially in the coastal regions, through coastal flooding, erosion, and destruction of houses in vulnerable areas along the coast. The high wave attacks, erosion and flooding, forced the evacuation of hundreds of families in each affected District. The ocean state, weather, and storm surge forecasts were well in place^8^. The storm surge predicted with the operational forecast system is about 0.15 m at the Chellanam coast and showed no coastal inundation in the present operational storm surge inundation forecast system. Moreover, the impact period at the Chellanam coast corresponded with the low tide. Despite these conditions, the coast was severely flooded with wave overwash that highlighted the complex coastal wave dynamics and its interaction with the underlying bathymetry.

The flooding at Chellanam is purportedly due to the infragravity waves and the wave setup that cause a resultant increase in the mean water level at the coast, facilitating wave overwash and inland inundation. The infragravity or long frequency waves associated with the incoming short wave bands^9^ elevate the total wave runup. As the infragravity waves increase the coastal surface water elevation, they might significantly contribute towards extending the coastal inundation during the wave overwash under cyclone conditions. The coastal water elevations are also increased due to the wave setup formed under breaking waves, where the cross-shore gradient in the radiation stress results in the rise of the mean water level at the coast^10^. The infragravity waves are not resolved by the operational forecast system for coastal inundation during the cyclone. Even though the forecast system includes the effect of wave radiation stress, a coarser grid resolution of ~ 100 m at the shoreline has failed to simulate the coastal inundation at Chellanam during the TC Tauktae. A forecast system for coastal inundation that incorporates the complex coastal wave hydrodynamics is very much needed in places like Chellanam, Kerala, where the high waves create frequent coastal inundations and destruction to livelihood. Hence, the present study attempts to predict wave-induced coastal inundation during the TC Tauktae to explore the possibility of an inundation forecast system for Chellanam. We used a combination of WAVEWATCHIII and XBeach models for the study.

## Tropical cyclone Tauktae

**Figure 1:**
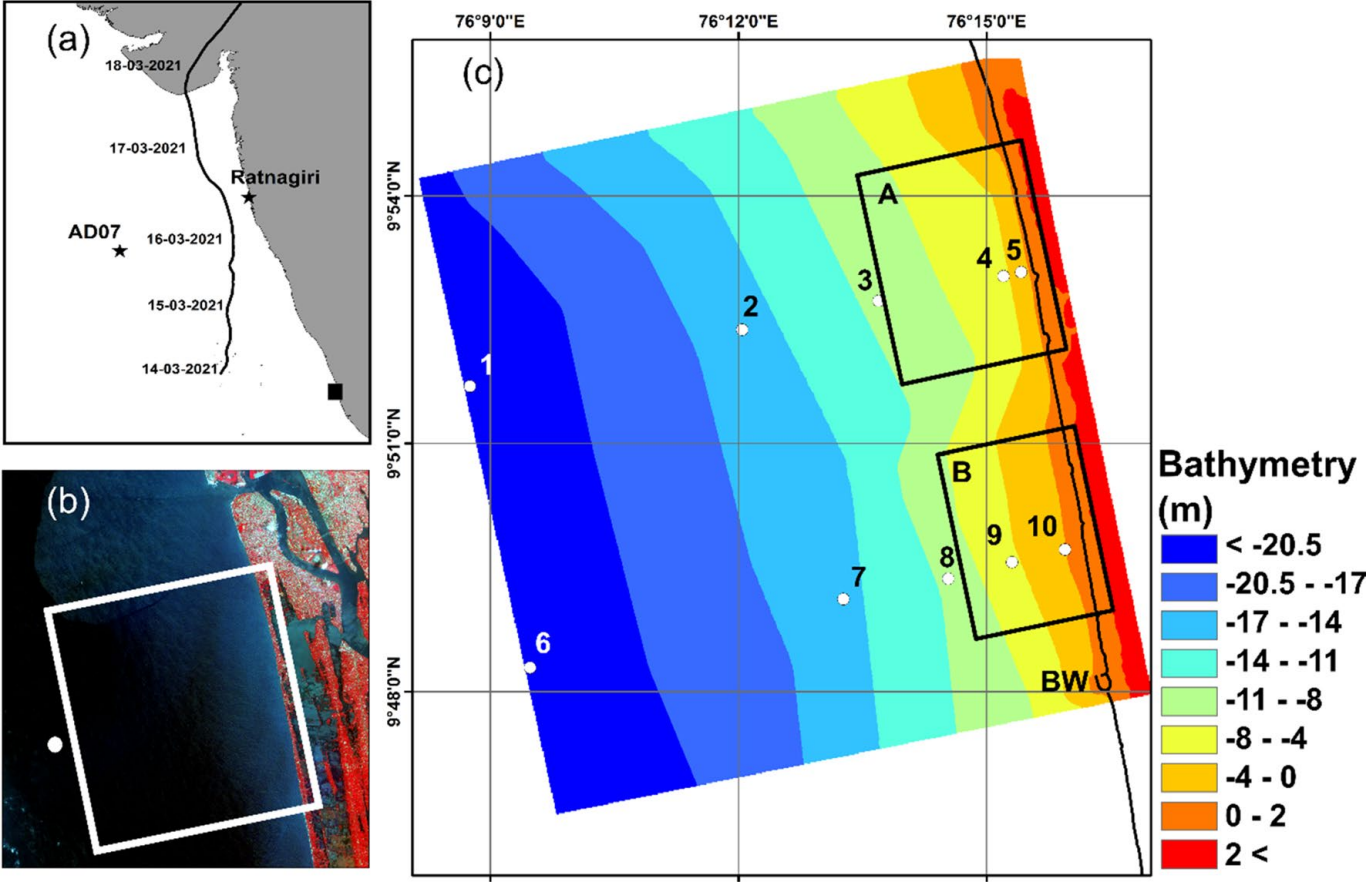
Study region showing (**a**) Arabian sea overlaid with the track of TC Tauktae, location of wave rider buoy AD07 and Ratnagiri used to validate WW3 is marked, (**b**) LISS-IV image of Chellanam region, location of time series Wave Watch III data used to force the XBeach model is shown as white circle; (**c**) Bathymetry of the domain used to simulate the nearshore wave dynamics using XBeach model, BW is the break water, the inset demarcates regions as A and B and the point locations 1 to 10 are used to estimate H_ln_ [We have used licensed version of ArcGIS desktop version 10.5 available at Space Applications Centre to prepare this figure, http://www.esri.com/].

TC Tauktae was the first very severe cyclonic storm over the north Indian Ocean in 2021 and the most intense cyclone of the AS during the satellite era (1961–2021) after the Kandla cyclone in 1998. A well-marked low-pressure area formed over the southeast AS and adjoining Lakshadweep area on May 13, 2021. Under favourable environmental conditions, it concentrated into a depression over the Lakshadweep area in the morning of May 14, 2021, and intensified into a deep depression in the afternoon. The deep depression further intensified into cyclonic storm “Tauktae” at the same midnight of May 14 over the same region, which then intensified into a severe cyclonic storm and moved northward on May 15 (Fig. 1). Continuing to move nearly northwards, it intensified into VSCS in the early hours of May 16. It gradually started moving north-northwestwards from noon (1130 hours IST/0600 UTC) of May 16 and intensified rapidly into an extremely severe cyclonic storm in the early hours of May 17. After that, it entered a marginally unfavourable environment, weakened gradually and crossed the Saurashtra coast near latitude 20.8° N and longitude 71.1° E, close to the northeast of Diu during 2000–2300 hours IST of May 17, 2021 with a maximum sustained wind speed of 160–170 kmph gusting to 185 kmph. TC Tauktae caused adverse weather and damage over entire west coast states, Union Territories and Lakshadweep as it moved parallel to the west coast and crossed Gujarat.

## Study area

Chellanam is a coastal village located on the southwest border of the Ernakulam district. The coastal stretch of Chellanam village extends to about 15 km (Fig. 1). A total population of almost 16,000, mostly belonging to the working class and farming community, fishing, agriculture, aquaculture etc., with relatively modest or poor living conditions, are staying in the village. The major issue faced is coastal erosion and inundation, which has been creating serious havoc among the people due to the destruction and loss of houses constructed near the shore, especially during high swell events and monsoon. Recently the passage of TC Taukate badly affected the entire coastal belt of Chellanam. Huge waves overtopped the sea wall resulting in floods in the low-lying areas. Severe damages occurred to the houses, household items, vehicles and other infrastructure facilities. The adopted protection measures (Seawall and geotubes or a combination of these measures) all along the coast are inadequate to manage the erosion and inundation along the Chellanam coastal stretch. These protection structures were critically damaged in several places on the Chellanam coast, causing overtopping during high waves^11^.

## Data and methodology

### Bathymetry

The present study has used a coastal high-resolution blended bathymetry merged with a topographic database. The bathymetry data is a blend of in-situ data (hydrographic charts, surveyed data from ships) for coastal regions and the General Bathymetric Chart of Ocean (GEBCO) data of 30 m spatial resolution towards the offshore. The outlier filtering was performed using a 2-sigma of semi-variance value within a 9 × 9 kernel spatial running window to avoid the abnormal spatial spike on the blended bathymetry. The blended coastal bathymetry is accurate, with an RMSE of 0.66 m in shallow waters (up to 60 m depth), which is essential to enhance the accuracy of the coastal modelling and inundation simulations. A high-resolution (5 m) Airborne Lidar Terrain Mapping (ALTM) topography data with 30 cm vertical accuracy up to 2 km from the coast and Cartosat-1 DEM (CartoDEM) data beyond 2 km were used as sources of the land elevation along the coastal zones of the study area. All these datasets were corrected to a common MSL datum.

### Models used

#### WAVEWATCH III

WAVEWATCH III (WW3) version 6.07, with ST4 parameterization scheme^12^ and with 4 grid mosaic a global grid of 1° spatial resolution, two regional grids (Indian Ocean (0.5) and northern Indian Ocean (0.25°)) and a coastal grid (0.04°)) for the Indian Ocean region was forced with ECMWF wind fields and generated the wave fields^13^. The model uses a spectral grid that consists of 29 frequencies and 36 directions. The wave spectrum extracted along the location shown in Fig. 1 is used as the open boundary condition for the 2D XBeach model.

#### XBeach model

The XBeach surf beat mode resolves the short wave variations on the wave group scale and allows the representation of long waves^14^. A dependent wave-action balance equation is solved using the dissipation model to derive the wave group forcing^15,16^. The momentum after breaking is represented by a roller model^17^. The associated radiation stress gradients exert force on the water column, thus representing the setup, wave-driven currents and longwave swash. The nonlinear shallow water equations solve the long-period waves and unsteady currents^14^. The mathematical description of the model and the numerical schemes involved are detailed in^15,16^.

A report of the under-prediction of longwave runup^18^ prompted subsequent improvements in the XBeach with a single direction scheme to better predict the short wave groupiness. The performance of the XBeach in predicting long-period waves was evaluated for the Hambantota Port in Sri Lanka and observed accurate prediction of long waves in the open domain^19^. Although using stationary wave conditions, the performance of the XBeach in simulating coastal erosion has been evaluated for the Indian coastal region by^20,21^.

The XBeach model is configured in 2D, where we have used varying grid resolution in the across-shore direction with 20 m resolution set to the coastal region, and the longshore grid resolution is kept constant at 20 m. The high-resolution blended bathymetry and topography (“Bathymetry” section) are used to create the domain shown in Fig. 1. As the present study focuses on coastal inundation, we have excluded sediment transport and morphological updating. The directional wave spectrum from 13 to 17 March 2021 extracted for the location shown in Fig. 1b from WW3 is used to force the XBeach model along with the predicted tidal elevation using the Global Tide Model of MIKE21 toolbox developed by DTU Space^22^.

The significant wave height of the longwave (H_ln_) and the short wave (H_sh_) is computed from the time series information of model output written for point locations marked from 1 to 10 in Fig. 1. The energy spectrum is obtained from the variance of the time series surface elevation filtered within the frequency range of infragravity waves (0.005–0.04 Hz) at the locations and the zero-order moment of the energy spectrum (*m*_0_) is used to estimate *H*_*ln*_^14,19,23^ as

1 $$H_{ln} = 4\sqrt {m_{0} }$$

## Results and discussions

**Figure 2:**
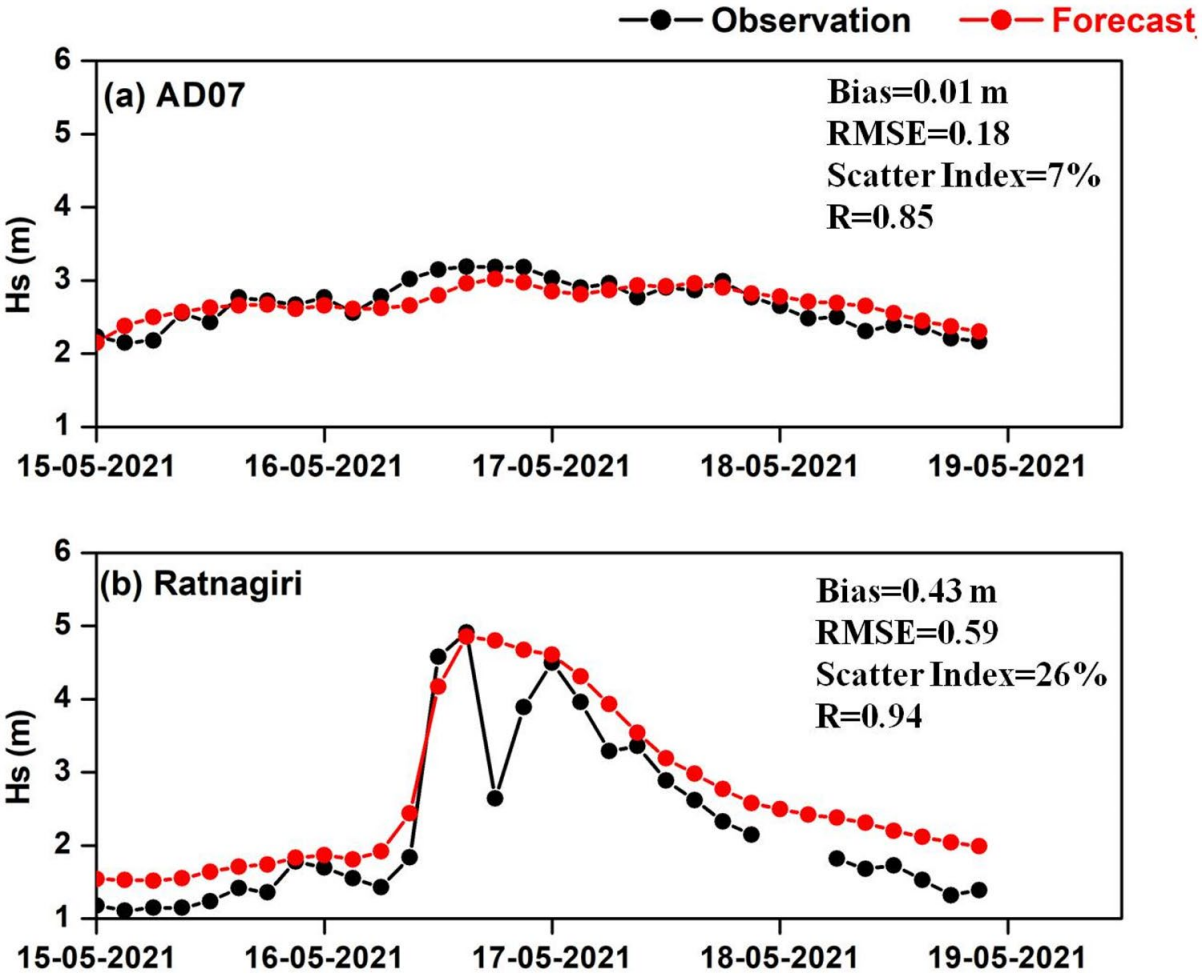
Validation of WW3 significant wave height forecast with buoy observations (**a**) offshore (**b**) coastal.

The inundation of the coastal area along the Chellanam hamlet on the southern coast of India during the TC Tauktae was in the limelight as several households, roads and public facilities were severely affected. The XBeach model was applied in surfbeat mode to simulate the wave conditions from May 13 to May 17, 2021. Figure 2 shows the significant wave height (Hs) validation at an offshore and coastal buoy location (Fig. 1a) during TC Tauktae. It indicates the ability of the operational WW3 wave model to accurately simulate the cyclone-induced high waves in the area of interest, thereby ensuring the correctness of the wave boundary conditions given to the XBeach model.

**Figure 3:**
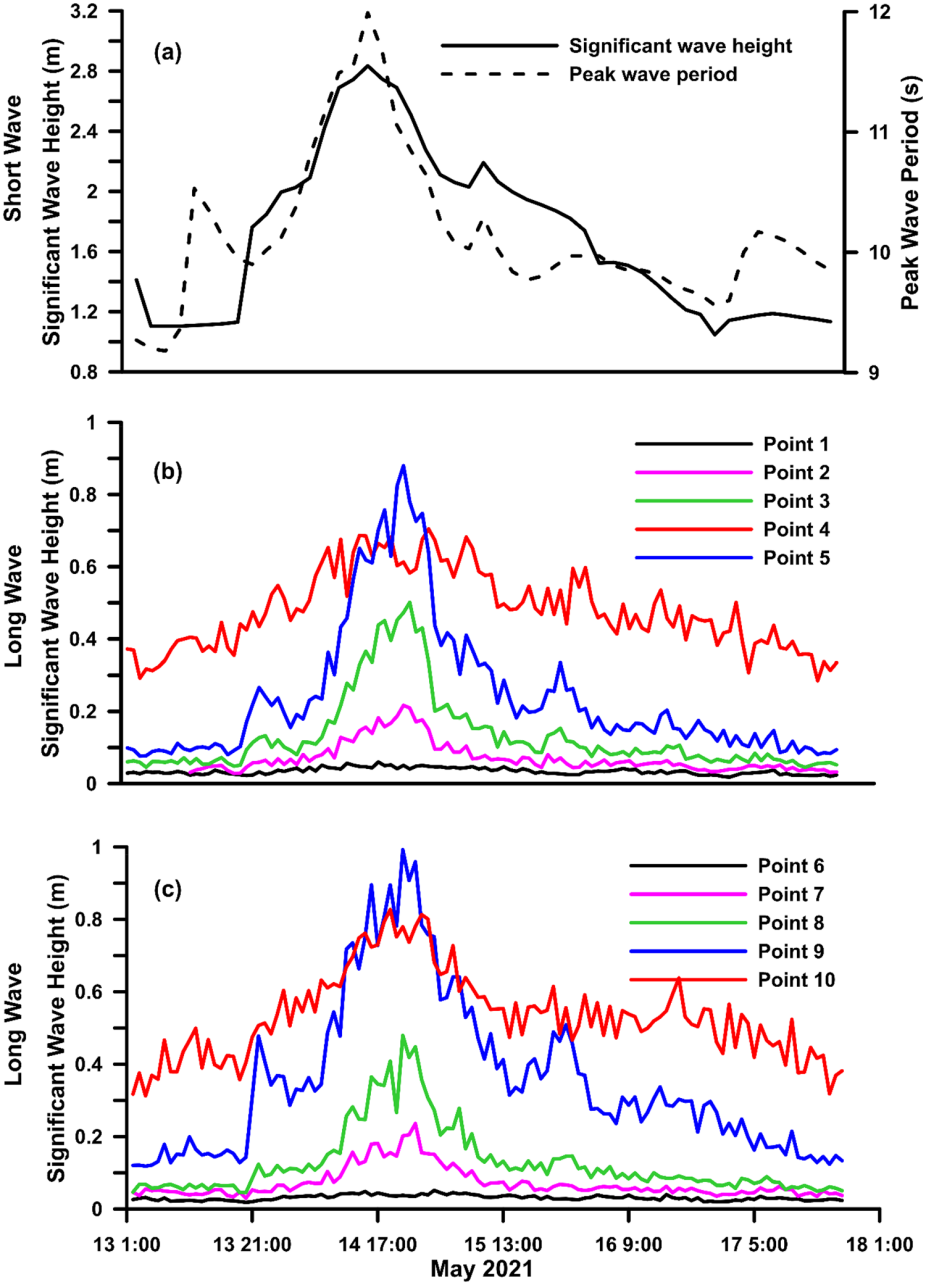
(**a**) Shot wave parameters at the offshore boundary; (**b**) Significant wave height at points 1 to 5 (Fig. 1); (**c**) Significant wave height at points 6 to 10 (Fig. 1).

The significant wave height of the longwave component (H_ln_) is estimated as described in section “XBeach model” for the point locations shown in Fig. 1c. Five locations are taken for each region corresponding to offshore bathymetry contours of − 15, − 10 and − 5 m near the shoreline. Figure 3b,c show the time series H_ln_ estimated for the point locations at regions A and B, and the offshore wave condition is plotted in Fig. 3a. A notable increase in the H_ln_ can be observed from 14:00 h on May 14 until 10:00 h on May 15, 2021, specifically at point locations near the coast. Hln peaks at point locations in both regions correspond to a − 5 m bathymetry contour. The relative increase in H_ln_ corresponds to the time when high waves (H_sh_, Fig. 3a) generated by TC Tauktae reached the coast of Chellanam. The amplitude of the long wave is approximately proportional to the height of the incident short wave and independent of the period^24^.

**Figure 4:**
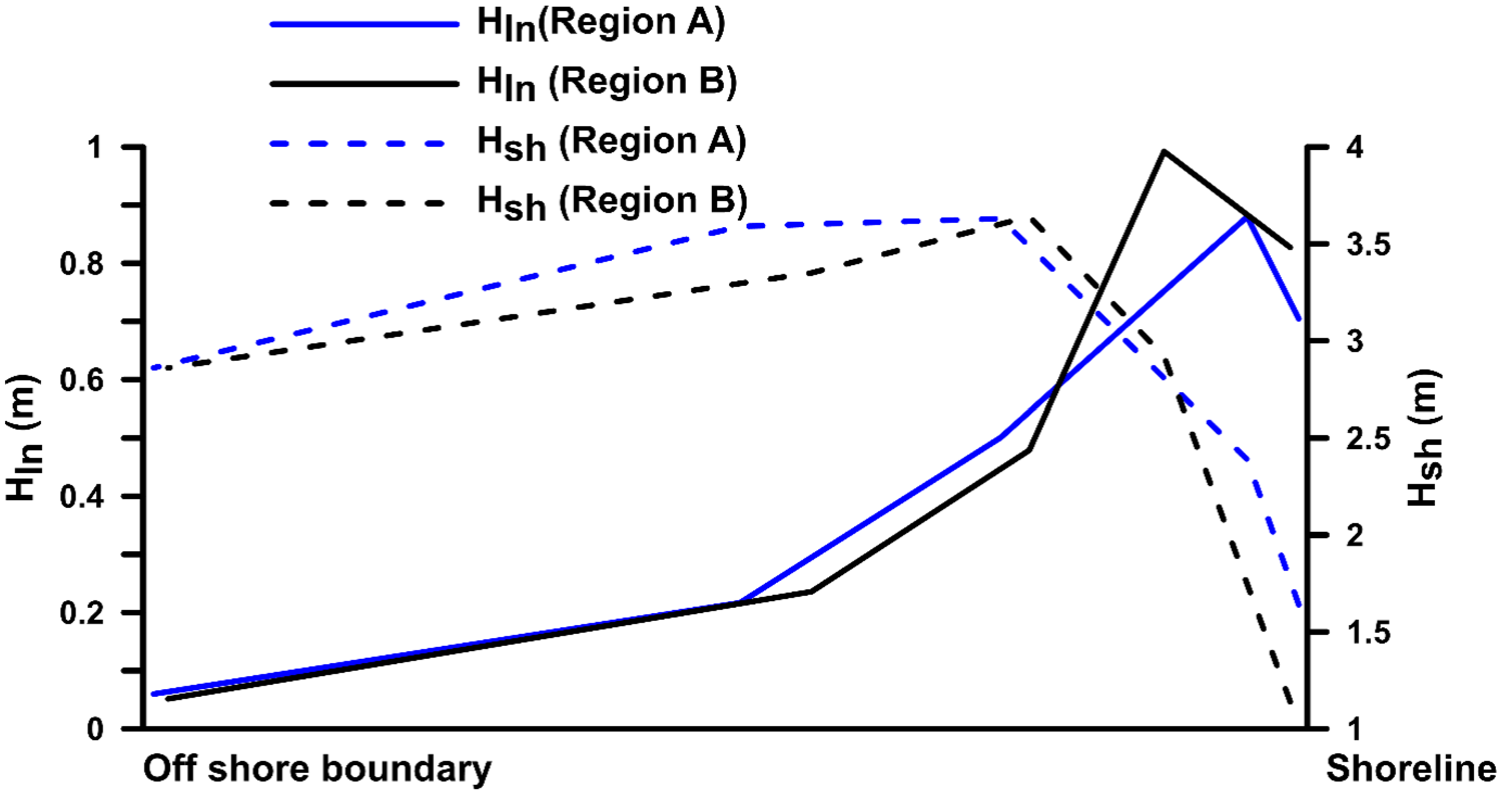
The change in the significant wave height of H_sh_ and H_ln_ at region A and B from offshore boundary to the shoreline.

Figure 4 shows the change in the significant wave height of H_sh_ and H_ln_ for regions A and B, from the offshore boundary to the coast during the highest wave event of the cyclone impact. While approaching the coast, the energy of the short wave gets dissipated, and the wave height is reduced. In contrast, the wave height of the longwave component increases from negligible height at the boundary toward the coast. In both regions, the peak of H_ln_ at − 5 m is observed to reduce as the wave approaches the shoreline. The significant wave height of H_ln_ at the shoreline of region A is about 0.7 m, while at the shoreline of region B, the H_ln_ is about 0.8 m. Ruju et al.^25^ observed the energy of the infragravity waves to increase at the outer surf zone, where the gradient in the radiation stress balance the nonlinear energy transfer from swell to infragravity waves. The increase in the infragravity waves is limited at the outer surf zone, where the dissipation starts towards the shoreline. Infragravity wave growths in the inner surf zone can be higher along gently sloping bathymetry due to long propagation time^26^. The coastal slope at region B is gentle compared to region A (Fig. 6) and shows an increased infragravity wave height near the coast.

**Figure 5:**
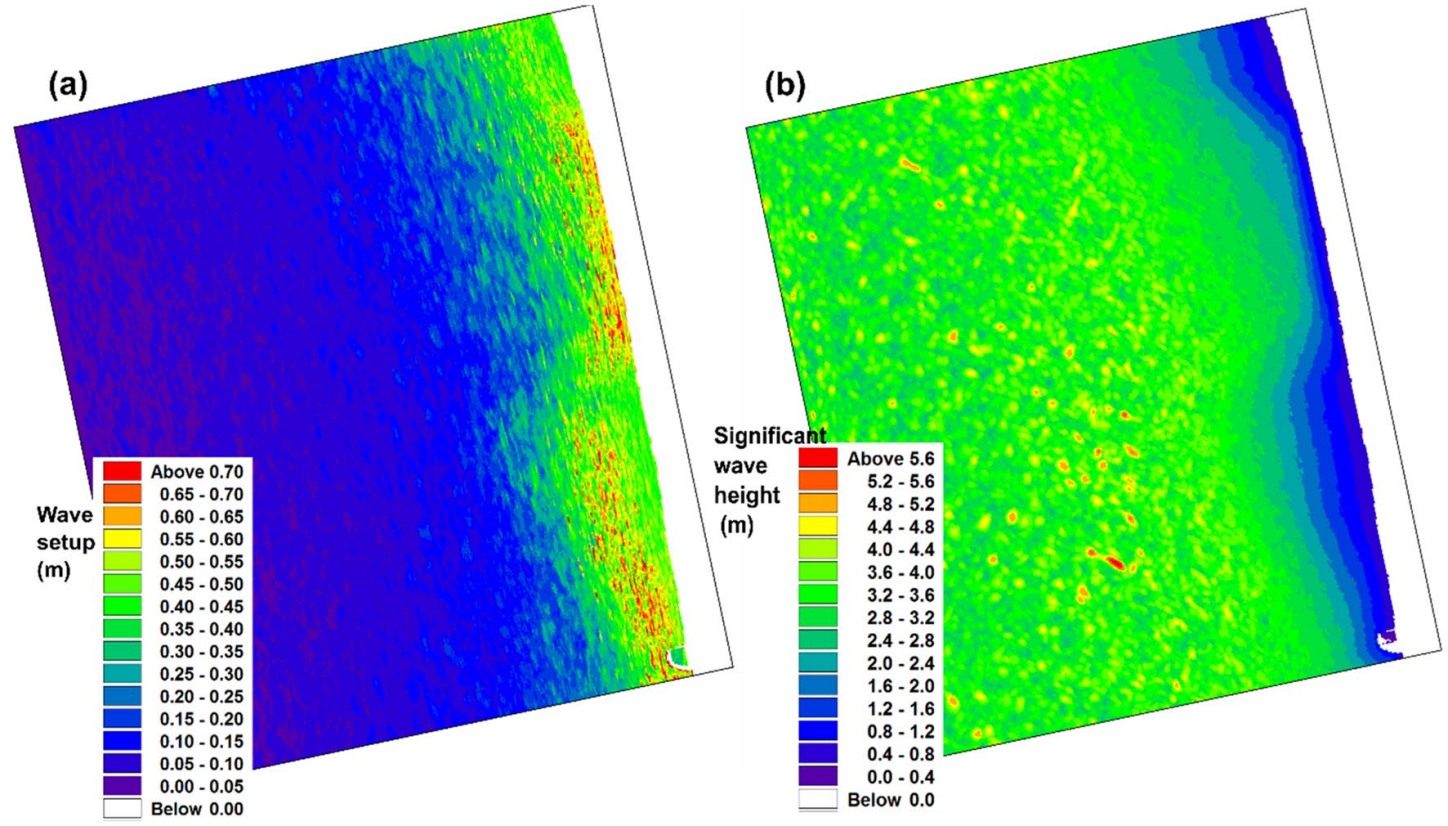
Simulated maximum (**a**) wave setup and (**b**) significant wave height (short wave) during the period of TC Tauktae at Chellanam.

**Figure 6:**
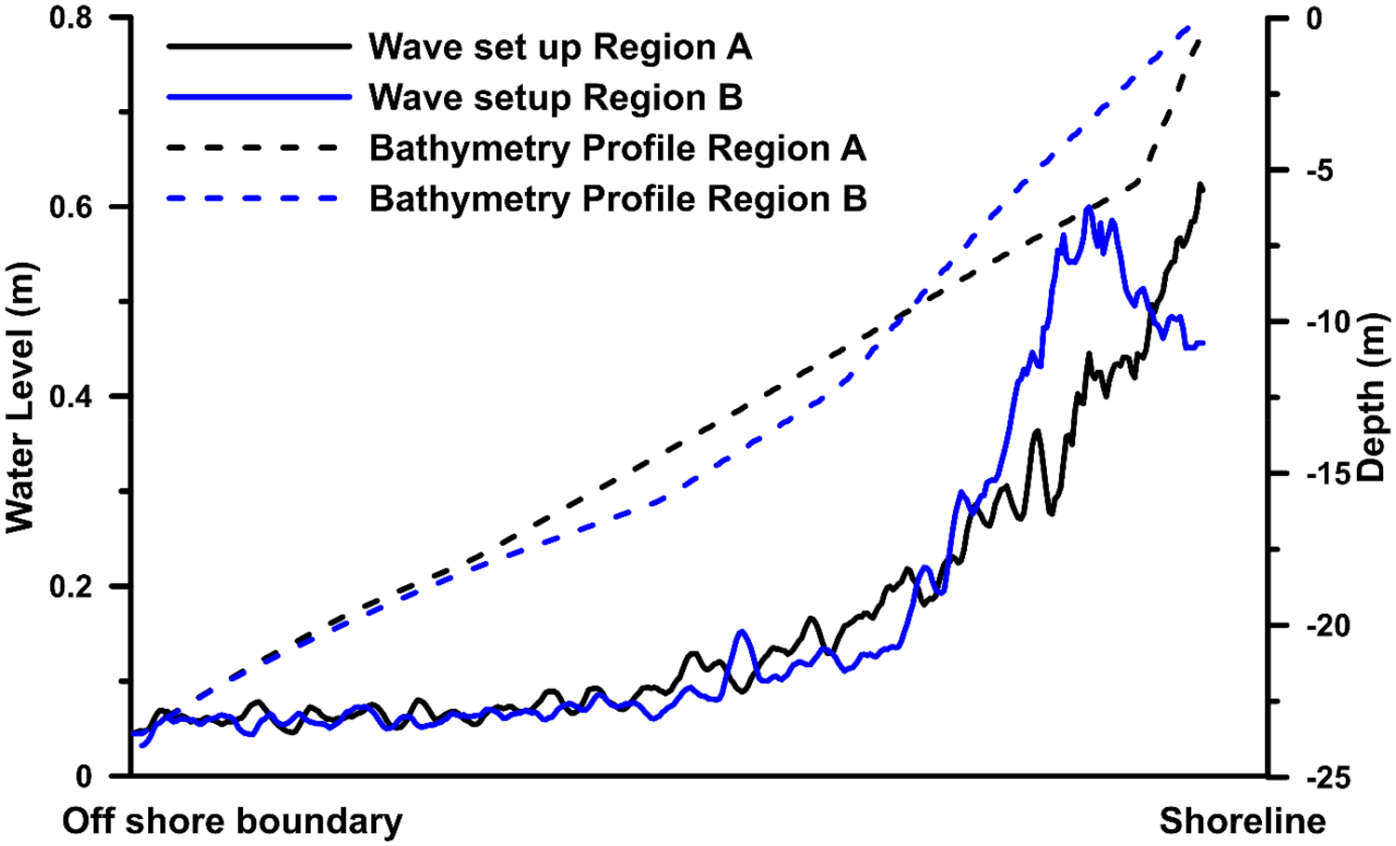
Maximum water level due to wave setup at region A, B, along with the corresponding bathymetry profiles.

The momentum of the waves is transferred to the water column in the surf zone, which leads to an increase in the water level called the wave setup. The water level from May 14, 14:00 h to May 15, 10:00 h, corresponding to the peak storm, is analyzed to obtain the maximum water level at each grid and is shown in Fig. 5a, and the significant wave height obtained with the same procedure is shown in Fig. 5b. Along the coastal zone, the maximum significant wave height shows spatial variability, where the coastline in region A is impacted with higher waves compared to the region marked as B. Spatial variation in the maximum water level due to wave setup (Fig. 5a) is prominent along the coast. The water levels are high on the northern coast (region marked as A), and in the region marked as B, the highest water level falls far from the coast. Figure 6 shows the average maximum water level (Fig. 5) estimated along 10 cross-shore profiles at regions A and B, and the corresponding cross-shore bathymetry profiles are plotted. In region A, the cross-shore bathymetry from − 6 m to the shoreline has a sudden decrease in depth, forming a steep slope of 0.22. At the same time, the bathymetric slope at region B is relatively steep, between − 10 and − 6 m, which is located away from the coast. From − 6 m to the shoreline, the bathymetry shows a gentle slope of 0.08 in region B. The water level at region A is steep towards the coastal region; the elevation reaching a maximum of over 0.6 m near the shoreline^27^ established an empirical relationship for wave setup that is proportional to the slope. It can be observed that the wave setup is steep at region A, where the bathymetry profile forms a steep slope. Whereas the water elevation at region B reaches a maximum of about 0.6 m at a distance of about 1 km from the shoreline, and then it gradually drops to around 0.4 m at the shoreline. As observed from the bathymetry profile of region B, the slope is steep away from the coast between − 10 to − 6 m, which possibly has increased the wave setup. Moreover, towards the coast, the slope reduced with a gradual decrease in the surface water elevation.

**Figure 7:**
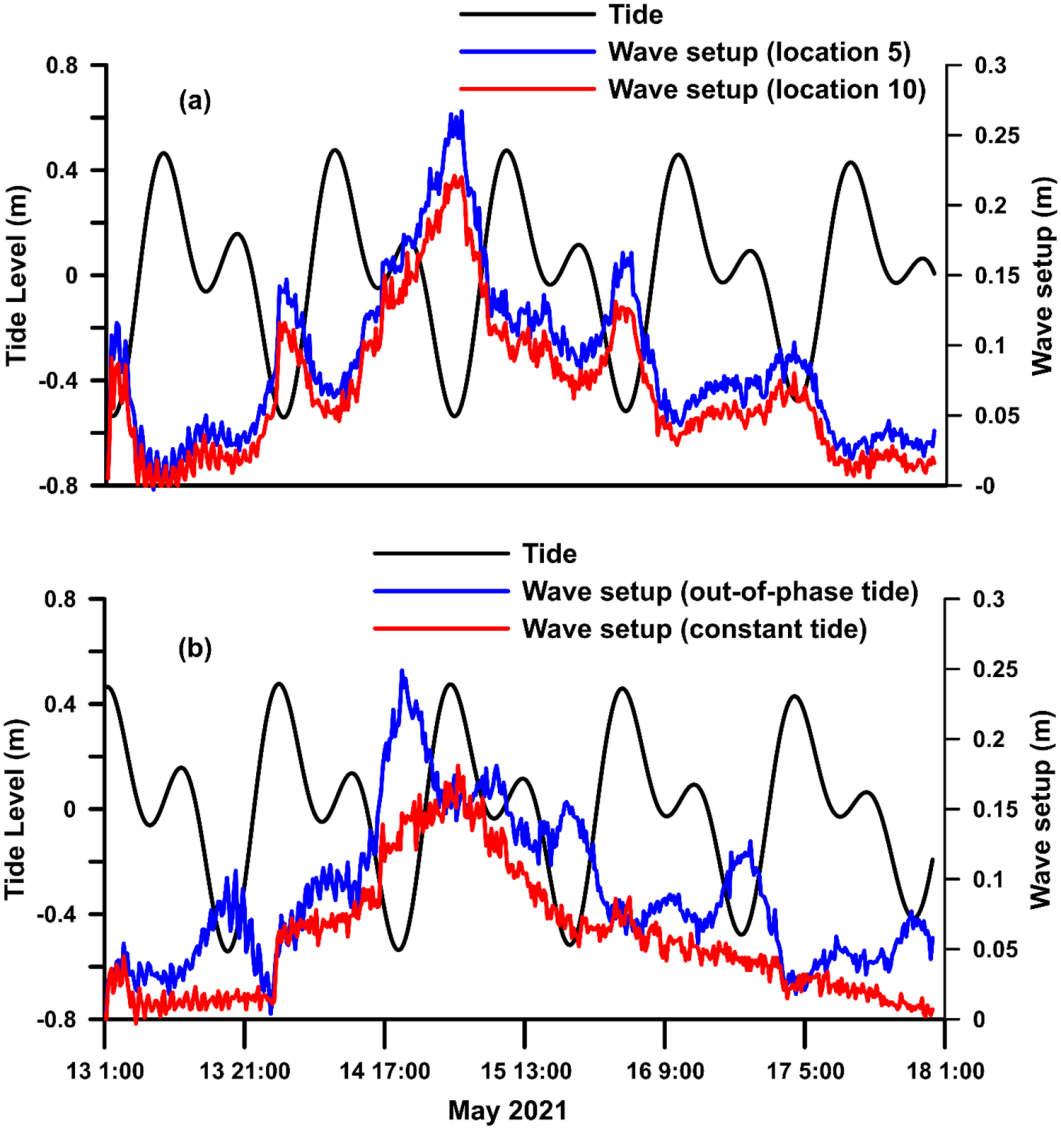
(**a**) Predicted tide at Chellanam and wave set up averaged over 15 min at locations 5 and 10 of regions A and B, respectively. (**b**) Experimental simulation with the out-of-phase tide and constant tide of 0.4 m at region A.

The impact of the TC Tauktae at the Chellanam coastal region occurred during low tide, which may have increased the wave setup. From the time series water elevation at point locations 5 and 10, the average is estimated for 15-min intervals and is plotted in Fig. 7a along with the tidal condition. During the storm wave conditions, the peak in wave setup is concomitant to the low tide. A small peak in wave setup is also observed during the non-storm condition coinciding with the low tide condition. We carried out two experimental simulations to understand the effect of tidal conditions on wave setup. In the first simulation, the model is forced with the out-of-phase tide, and in the second simulation, a constant tide of 0.4 m is given while retaining the same wave boundary parameters.

The out-of-phase tide and corresponding averaged surface water elevation at station 5 are plotted in Fig. 7b, where it can be observed that the peak in wave setup during the storm wave shifted in time to be concurrent with the low tide. The surface water elevation simulated with the constant tide is also shown in Fig. 7b. The peak wave setup with the constant tide has decreased to 0.17 m. In comparison, the wave setup simulated with tidal variation has peak values of more than 0.25 m which corresponds to the low tidal condition^28^ give a plausible reason that with increased water depth during high tide, large waves reach the shore without breaking, resulting in a reduced height of wave setup. During low to mid-tide, the wave setup gets pronounced due to nearshore wave breaking. The shoreline of Chellanam is protected with a seawall, and due to the presence of steep coastal bathymetry, during high tide, the waves may reach the seawall without breaking, while the low tide favours nearshore wave breaking that induces wave setup and elevated water level at the coast.

**Figure 8:**
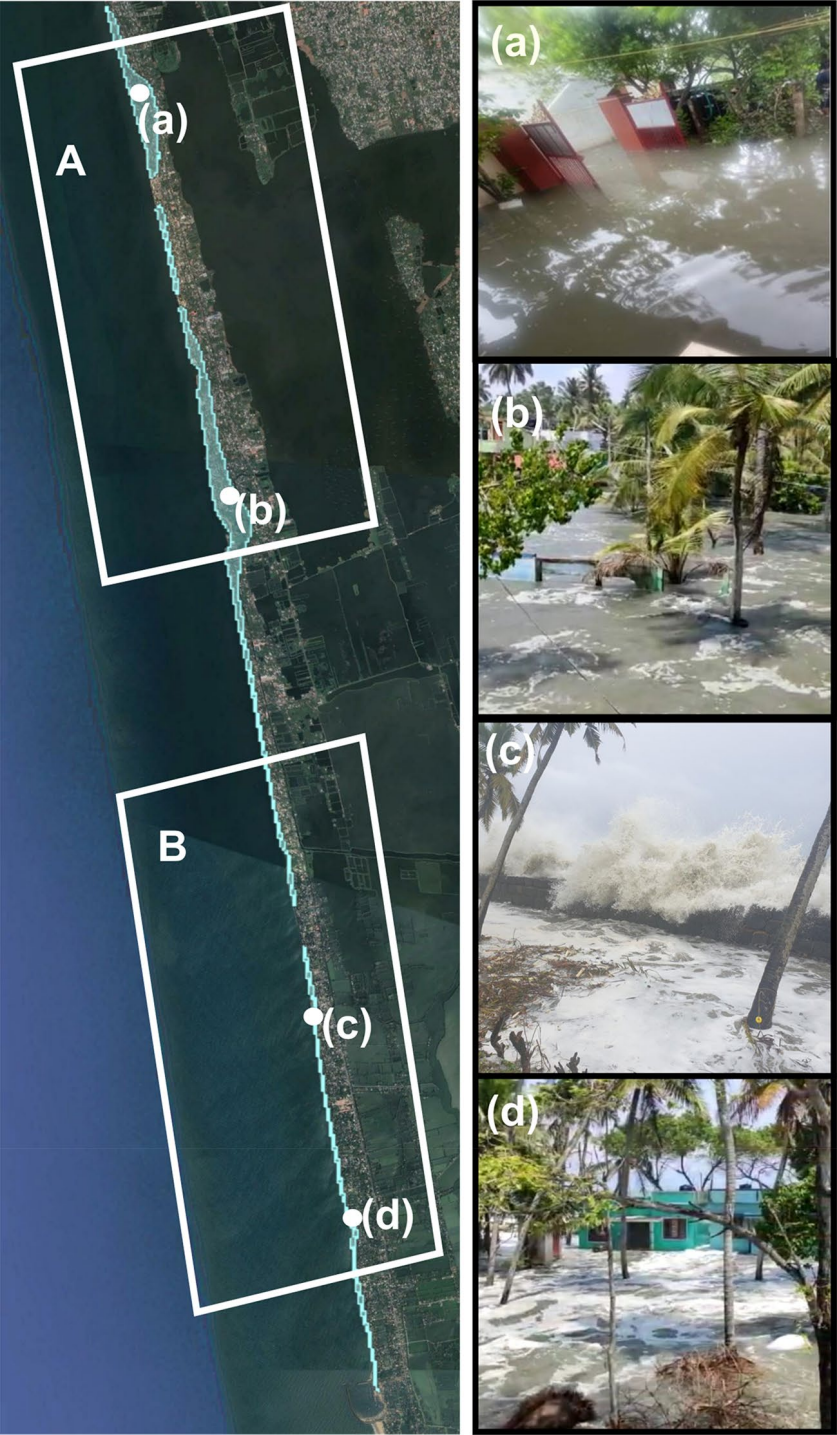
Simulated coastal inundation at Chellanam over Google Earth images. The point locations shown are (**a**) Cheriyakadavu, (**b**) Kannamali, (**c**) Velankanni, (**d**) Kandakkadavu and the corresponding photographs of inundation are shown in the right panel.

Figure 8 shows the maximum inundation extent during the period overlaid on Google Earth. Even though the waves overtopped and inundated the entire coastline, the landward inundation is maximum to the northern part of the domain. The XBeach model in surfbeat mode has simulated the long period infragravity waves that increased its height as the wave propagated to the shoreline and had a peak value of more than 0.7 m near the coast. The maximum surface water elevation at the shoreline for region A due to the wave setup was 0.7 m. The combined effect of infragravity waves and wave setup increased the coastal water elevation to about 1.5 m, over which the storm waves acted along the coast, overtopped the coastal structures and inundated the low-lying regions. The inland inundation reached about 300 m in the northern part. Reports during the TC Tauktae have confirmed the inundation of the coastal road around 300 m away from the shoreline at places (https://www.thehindu.com/news/national/kerala/cyclone-tauktae-chellanam-continues-to-reel-under-flooding-people-shifted-to-relief-camps/article34565220.ece).

## Conclusions

The simulation carried out to study the inundation of Chellanam emphasizes the contribution of infragravity waves and wave setup on the overtopping of the waves inundating the coastal regions that are often ignored in the operational framework of coastal inundation during cyclone conditions. The coastal inundation at Chellanam is important, as the storm surge during the cyclone was negligible, as observed from the tide station data at the adjacent Cochin Port and the time of high wave impact corresponds to the low tidal conditions. Despite the above conditions, the inundation at Chellanam has severely affected the settlements. The waves severely damaged many houses, and overtopped water flushed past beach road and caused waterlogging even at those on the eastern side of the road.

The bathymetry slope has crucially controlled the wave setup elevation, which peaked at about 0.7 m at the shoreline with steep bathymetry profiles. The temporal variability is influenced by the incoming short and long waves and tidal conditions. The simulation results show that the wave setup has peak elevation during the low tide time. Experimental simulation with constant high tide conditions significantly reduced the wave setup elevation, showing the effect of low to mid-tide conditions in enhancing the wave setup elevation. The combined impact of short wave, longwave component and wave setup on the maximum runup extent is modulated by the steepness of the bathymetry and the tidal conditions. The peak in the longwave and wave setup corresponded to the high waves from the TC Tauktae, resulting in wave overwash that caused severe flooding, and the coastal residences at Chellanam were severely affected. The study also envisages the modelling framework to include the longwave component and the wave setup for operational inundation forecast during the cyclone and the coastal flooding during the high swell waves or the Kallakadal phenomenon. The development of wave-induced inundation and erosion forecast systems for selected hot spots is the need of the hour as extreme waves may cause extreme damage to the coast, and in the anticipated climate change scenario, with increased storm surges; heavy rains and rising sea level, the impact on the coastal region will be extremely adverse.

## Data Availability

The mooring observations used in this article can be accessed upon request from INCOIS (https://incois.gov.in/portal/datainfo/drform.jsp ).

## References

[1] Masson-Delmotte V, Zhai P, Pirani A, Connors SL, Péan C, Berger S, Caud N, Chen Y, Goldfarb L, Gomis MI, Huang M, Leitzell K, Lonnoy E, Matthews JBR, Maycock TK, Waterfield T, Yelekçi O, Yu R, Zhou B, IPCC (2021). Summary for policymakers. Climate Change 2021: The Physical Science Basis. Contribution of Working Group I to the Sixth Assessment Report of the Intergovernmental Panel on Climate Change.

[2] Zhang W, Villarini G, Vecchi GA, Smith JA (2018). Urbanization exacerbated the rainfall and flooding caused by hurricane Harvey in Huston. Nat. Lett..

[3] Zhu L, Emanuel K, Quiring SM (2021). Elevated risk of tropical cyclone precipitation and pluvial flood in Houston under global warming. Environ. Res. Lett..

[4] Rehman S, Sahana M, Kumar P, Ahmed R, Sajjad H (2020). Assessing hazards induced vulnerability in coastal districts of India using site-specific indicators: An integrated approach. GeoJournal.

[5] Sahoo B, Bhaskaran PK (2018). A comprehensive data set for tropical cyclone storm surge-induced inundation for the east coast of India. Int. J. Climatol..

[6] Singh OP (2001). Has the frequency of intense tropical cyclones increased in the north Indian Ocean?. Curr. Sci..

[7] Unnikrishnan AS, Kumar KR, Fernandes SE, Michael GS, Patwardhan SK (2006). Sea level changes along the Indian coast: Observations and projections. Curr. Sci..

[8] Mandal AK, Ratheesh R, Pandey S, Rao AD, Kumar P (2020). An early warning system for inundation forecast due to a tropical cyclone along the east coast of India. Nat. Hazards.

[9] Bertin X (2018). Infragravity waver: From driving mechanisms to impacts. Earth Sci. Rev..

[10] Wu G, Shi F, Kirby JT, Liang B, Shi J (2018). Modeling wave effects on storm surge and coastal inundation. Coast. Eng..

[11] Restoration of Chellanam panchayath, Interim Report submitted to the Hon’ble Minister for Fisheries, Culture and Youth Affairs Government of Kerala, Kerala University of Fisheries and Ocean Studies (KUFOS), July (2021).

[12] Ardhuin F (2010). Semi empirical dissipation source functions for ocean waves. Part I: Definition, calibration, and validation. J. Phys. Oceanogr..

[13] Remya PG, Ranjan TR, Sirisha P, Harikumar R, Nair B (2020). Indian Ocean wave forecasting system for wind waves: Development and its validation. J. Oper. Oceanogr..

[14] Roelvink D, McCall R, Mehvar S, Nederhoff K, Dastgheib A (2018). Improving predictions of swash dynamics in XBeach: The role of groupiness and incident-band runup. Coast. Eng..

[15] Roelvink JA, Reniers A, van Dongeren A, van Thiel DVJ, McCall R, Lescinski J (2009). Modelling storm impacts on beaches, dunes and barrier islands. Coast. Eng..

[16] Roelvink JA, Reniers A, van Dongeren A, van Thiel DVJ, Lescinski J, McCall R (2010). XBeach Model Description and Manual.

[17] Roelvink, D. & Reniers, A. *A Guide to Modeling Coastal Morphology Advances in Ocean Engineering* (World Scientific, ISBN: 978-981-4304-25-2, 2011).

[18] Stockdon HF, Holman RA, Howd PA, Sallenger AH (2006). Empirical parameterization of setup, swash, and runup. Coast. Eng..

[19] Guo L, Ma X, Dong G (2021). Performance accuracy of surfbeat in modelling infragravity waves near and inside a harbour. J. Mar. Sci. Eng..

[20] Ratheesh R, Agrawal R, Remya PG, NagaKumar KCV, Demudu G, Rajawat AS, Nair B, Rao KN (2018). Modelling coastal erosion: A case study of Yarada beach near Visakhapatnam, east coast of India. Ocean Coast. Manag..

[21] Ratheesh, R. *et al*. A numerical modelling approach for beach erosion forecast during the southwest monsoon season. *J. Earth Syst. Sci*. (2022) **(accepted)**.

[22] Cheng, Y. & Andersen, O. B. Improvement in global ocean tide model in shallow water regions. Poster, SV.1-68 45 (OSTST, Lisbon, Oct. 18–22, 2010).

[23] Lashley CH, Bertin X, Roelvink D, Arnaud G (2019). Contribution of infragravity waves to runup and overwash in the Pertuis Breton embayment (France). J. Mar. Sci. Eng..

[24] Munk W (1949). Surf beat. Eos transactions. AGU.

[25] Ruju A, Lara JL, Losada IJ (2012). Radiation stress and low-frequency energy balance within the surf zone: A numerical approach. Coast. Eng..

[26] De Bakker ATM, Tissier MFS, Ruessink BG (2016). Beach steepness effects on nonlinear infragravity-wave interactions: A numerical study. J. Geophys. Res. Oceans.

[27] Stockdon HF, Thompson DM, Plant NG, Long JW (2014). Evaluation of wave runup predictions from numerical and parametric models. Coast. Eng..

[28] Xie D, Zou QP, Mignone A, McRae JD (2019). Coastal flooding from wave overtopping sea level rise adaptation in the northeastern USA. Coast. Eng..

